# Evaluating equity across the continuum of care for maternal health services: analysis of national health surveys from 25 sub-Saharan African countries

**DOI:** 10.1186/s12939-023-02047-6

**Published:** 2023-11-17

**Authors:** Firew Tekle Bobo, Augustine Asante, Mirkuzie Woldie, Angela Dawson, Andrew Hayen

**Affiliations:** 1 Department of Public Health, Institute of Health Sciences, Wollega University, Nekemte, Ethiopia; 2 School of Public Health, Faculty of Health, University of Technology Sydney, Sydney, Australia; 3 Department of Health Policy and Management, Jimma University, Jimma, Ethiopia; 4 Fenot Project, Department of Global Health and Population, Harvard T.H. Chan School of Public Health, Addis Ababa, Ethiopia; 5 School of Population Health, University of New South Wales, Sydney, Australia; 6 Department of Health Systems and Health Economics, Bahir Dar University, Bahir Dar, Ethiopia

**Keywords:** Equity, Socioeconomic factors, Maternal health, Continuum of care, Sub-Saharan Africa

## Abstract

**Background:**

Ensuring access to the continuum of care for maternal, neonatal, and child health is an effective strategy for reducing maternal and child mortality. We investigated the extent of dropout, wealth-related inequalities, and drivers of inequality in the continuum of care for maternal health services in sub-Saharan Africa.

**Methods:**

We analysed Demographic and Health Surveys (DHS) conducted between 2013 and 2019 across 25 sub-Saharan African countries. We defined the continuum of care for maternal health services as women who had received at least four ANC contacts (ANC 4 + contacts), skilled care at birth, and immediate postnatal care (PNC). We used concentration index to estimate wealth-related inequalities across the continuum of care. Multilevel logistic regression models were used to identify predictors of inequality in completing the continuum of care.

**Results:**

We included data on 196,717 women with the most recent live birth. About 87% of women reported having at least one ANC contact, but only 30% of women received the recommended care package that includes ANC 4 + contacts, skilled care at birth, and PNC. The proportion of women who had completed the continuum of care ranged from 6.5% in Chad to 69.5% in Sierra Leone. Nearly 9% of women reported not having contact with the health system during pregnancy or childbirth; this ranged from 0.1% in Burundi to 34% in Chad. Disadvantaged women were more likely to have no contact with health systems and less likely to have the recommended care package than women from wealthier households. Women with higher education levels, higher exposure to mass media (radio and TV), and higher household wealth status had higher odds of completing the continuum of care.

**Conclusions:**

Persistent and increasing inequalities were observed along the continuum of care from pregnancy to the postnatal period, with socioeconomically disadvantaged women more likely to drop out of care. Improving access to and integration of services is required to improve maternal health. Initiatives and efforts to improve maternal health should prioritise and address the needs of communities and groups with low coverage of maternal health services.

## Introduction

Ending preventable maternal mortality continues to be a persistent challenge worldwide, especially in low- and lower-middle-income countries (LLMICs) [1, 2]. Women in sub-Saharan Africa, in particular, have a significantly higher risk of dying from causes related to pregnancy and childbirth than in any other region, accounting for 70% of all maternal deaths in 2020 globally [3].

Over the past two decades, many healthcare reforms have been implemented to address healthcare needs and strengthen health systems globally [4]. In low- and middle-income countries, these reforms have been aimed at improving equity and providing infrastructure, with particular emphasis on reducing maternal and child mortality [4, 5]. However, these changes have not always brought anticipated reductions in maternal mortality. For example, from 1990 to 2022, global coverage of one or more ANC contacts increased from 65 to 88%, ANC 4 + contacts from 37 to 66%, and births attended by skilled providers from 57 to 86% [6, 7]. These gains were mainly observed in developing regions. Yet maternal mortality has remained high with no progress since 2015 and extreme inequalities — a woman’s lifetime risk of dying due to pregnancy and childbirth remaining more than 100 times higher in sub-Saharan Africa than in high-income countries [3].

The mismatch between the burden of mortality and coverage of services exposes a critical gap in the quality of care. Having access to services is one thing, but the quality of those services plays a crucial role – poor quality poses significant difficulties in improving maternal and child health (MCH) outcomes [8, 9]. For example, a study of 192 DHS found that institutional deliveries are only weakly associated with lower early neonatal mortality, suggesting that both utilization and quality of care need to be improved to further reduce maternal and child mortality [10].

Globally, as access to health services improved, the quality of care remained poor, becoming the key reason for the slow progress in MCH outcomes in many low-income countries [7, 8]. This is especially evident among disadvantaged populations, who frequently face limited access to health services and when they do manage to access the services, they often receive poor-quality care [3, 7]. Furthermore, in fragile, conflict-affected, economically, or politically unstable nations, both poor-quality and inaccessible healthcare services coexist, resulting in increased health disparities that disproportionately affect the most vulnerable populations [11].

Another critical factor impeding progress is the absence of consistent continuity of care from pregnancy through childbirth and the early postnatal period [12, 13]. Ensuring access to quality continuum of care for maternal, newborn, and child health across all countries and socioeconomic levels is critical to improving health outcomes and achieving the SDG target related to maternal health [12, 14].

However, access to health services is limited across many LLMICs, but more importantly, the services that do exist often lack integration and are weakly established, particularly during the critical phases of childbirth and the postnatal period [2, 15]. Furthermore, disparities in access to MCH services continue to exist within and between countries, along with factors such as geography, gender, religion, ethnicity, wealth, and other socioeconomic factors [16].

Addressing disparities in health requires generating evidence on inequality and determinants of inequality. In this paper, we examine inequities and attritions along the continuum of care for maternal health services. We assessed the extent of dropout along the continuum of care in these countries. We then explored within-country wealth-related inequalities across the continuum of care. We also identified predictors of inequality in the continuum of care and discussed the implications of our findings for health systems.

## Methods

### Data

We used the latest DHS data from 25 sub-Saharan African countries that were conducted between 2013 to 2019. We included surveys from Angola (2015), Burundi (2016–17), Cameroon (2018), Chad (2014–15), DRC (2013–14), Benin (2018), Ethiopia (2016), Ghana (2014), Guinea (2018), Kenya (2014), Lesotho (2014), Malawi (2015–16), Mali (2018), Namibia (2013), Nigeria (2018), Rwanda (2014–15), Senegal (2017), South Africa (2016), Zimbabwe (2015), Uganda (2016), Tanzania (2015), Zambia (2018), Gambia (2013), Togo (2013–14), and Sierra Leone (2019).

The DHS program uses standardised methods and model questionnaires to ensure uniformity and comparability of data collected across time and countries. Across all countries, the DHS uses standard survey design and sampling methods. The sampling strategies and methodology have been described elsewhere [17]. The sample used for this analysis includes all women who had at least one live birth during the five years preceding the respective surveys. We used the most recent live birth to reduce recall bias in this analysis.

### Measures

Our primary outcome variable of interest is the continuum of care for maternal health services. We defined the continuum of care for maternal health services as women who had received three services, including ANC 4 + contacts, skilled care at birth, and immediate PNC (within the first two days). We used ANC 4 + contacts because all the surveys in our analysis used the previous WHO standard for ANC visits, which recommends at least four ANC contacts.

We estimated coverage of ANC 4 + contacts as the proportion of women who had at least four ANC contacts with a skilled provider during their last pregnancy. We determined the adequacy of the content of ANC using five key interventions for which data were reported and consistently available in all DHS across all countries. These were iron supplements, blood pressure measurement, urine sample test, blood sample test, and tetanus protection at birth. We defined the early initiation of ANC as ANC contact before 12 weeks of gestation. We considered women who were unable to attend ANC or initiated later than 12 weeks of pregnancy to have delayed ANC initiation.

We defined coverage of skilled birth attendance (SBA) as a proportion of women who had professional care assistance during childbirth from qualified personnel, including a doctor, nurse, midwife, auxiliary midwife, or other cadres that each country considers skilled delivery attendants. We then estimated coverage of postnatal checks as a rate of women who had received care from a doctor, midwife, nurse, auxiliary midwife, or other cadres that each country considers skilled delivery attendants within the first two days of childbirth. Other cadres include, for example, the health extension workers in Ethiopia, and MCH aide in Tanzania.

### Covariates

We used a framework developed by the WHO Commission on Social Determinants of Health to explain determinants of inequality in completing the continuum of care. Household wealth index and educational status were considered to determine the socioeconomic position of women [16]. We used the wealth index variable constructed in the DHS using principal components analysis, which is based on ownership of selected household assets such as television (TV), radio, refrigerator, and vehicle; materials used for housing construction; and access to sanitation facilities and clean water. Household wealth index was ranked into quintiles from the poorest to the richest, depending on their level of wealth.

We categorized maternal education as (no education, primary, secondary, or higher). Women were asked if they had serious problems accessing health care for themselves when they were sick. We considered the distance to a health facility and financial constraints for treatment as potential barriers to accessing health services and categorised as – not a big problem or big problem. We considered exposure to media, which we categorized based on the frequency of reading newspapers, listening to the radio, and watching TV as not at all, less than once a week, and once a week or more. The type of place of residence was categorized as urban or rural. Our analysis also included maternal age (15 to 24, 25 to 29, 30 to 49) and parity (1 to 6).

### Statistical analysis

We used ArcGIS software V.10.7.1 to show the geographic distributions of maternal health services, including ANC 4 + contacts, skilled care at birth, and PNC at national and subnational levels.

We then analysed dropouts across the continuum of care for maternal health services. We calculated frequencies and proportions at individual points of contact along the care continuum—antenatal, childbirth and postnatal period. We followed the minimum recommended care pathway to assess the uptake of maternal health services at different contact points, including one ANC contact, ANC 4 + contacts, skilled care at birth, and immediate PNC. We used a decision tree to show the pathway choices women made at different points of care.

We used the concentration index (CCI) to estimate within-country wealth-related inequalities along the continuum of care [18]. Concentration indices are used to estimate relative inequality by calculating the distribution of one variable (e.g., having ANC 4 + contacts) over the other (wealth status) [18]. The index takes a value between—1 and + 1; an index of 0 indicates equality in completing the continuum of care. A positive CCI value indicates a pro-rich coverage of health variables (e.g., ANC 4 + contacts). A negative index implies a disproportionate concentration of the health variable among the poor.

We used multilevel logistic regression models to identify determinants of inequality in completing the continuum of care. The DHS program uses a multistage cluster sampling technique where participants in the survey are nested within Primary Sampling Units (PSU) across all countries [17]. Due to the application of complex survey methods to collect data (i.e., hierarchical data), we used a multilevel model to account for three-level data – women at level 1, PSU (clusters) at level 2 and country at level 3 [19, 20].

We started the model-building process with the unconditional model (a model containing no predictors), and then more complex models were built gradually. We used a Generalized Latent Linear Mixed Model (GLLMM) in Stata, which enabled us to adjust for the hierarchical nature of the data and the sampling weights [19]. We specified a 3-level model: level 1 variables included women (184,567) and their household factors; at level 2, we adjusted for clustering (14,590), and at level 3 we adjusted for country [25]. The initial weighted sample at level 1 consisted of 196,717 participants. However, due to missing data at the households or women level, the final weighted sample included in the multilevel regression model was reduced to 184,567 women.

The final model building was done in four stages. The initial step in the analysis involved entering community-level factors into the baseline model to examine association with the outcome variable. We considered community and individual level barriers to accessing healthcare, such as financial constraints to pay for treatment, long travel distances to attend facility, and place of residence. These variables were all significantly associated with the outcome variable and were retained for further analysis.

In the subsequent stage, socioeconomic factors were introduced to the model alongside the significant factors retained from the baseline model. Model 2 included wealth status, maternal education, and barriers to accessing healthcare factors. All of these variables were significantly associated with the outcome variable and were retained for further analysis. In Model 3, we retained all the variables from Model 2 and added maternal factors such as age and parity. All of these variables were significantly associated with the outcome. These factors were then retained in the final model (Model 4), which also included access to media variables. In each stage of the modeling process, we only included factors that were statistically significant (*p* < 0.05).

**Fig. 1:**
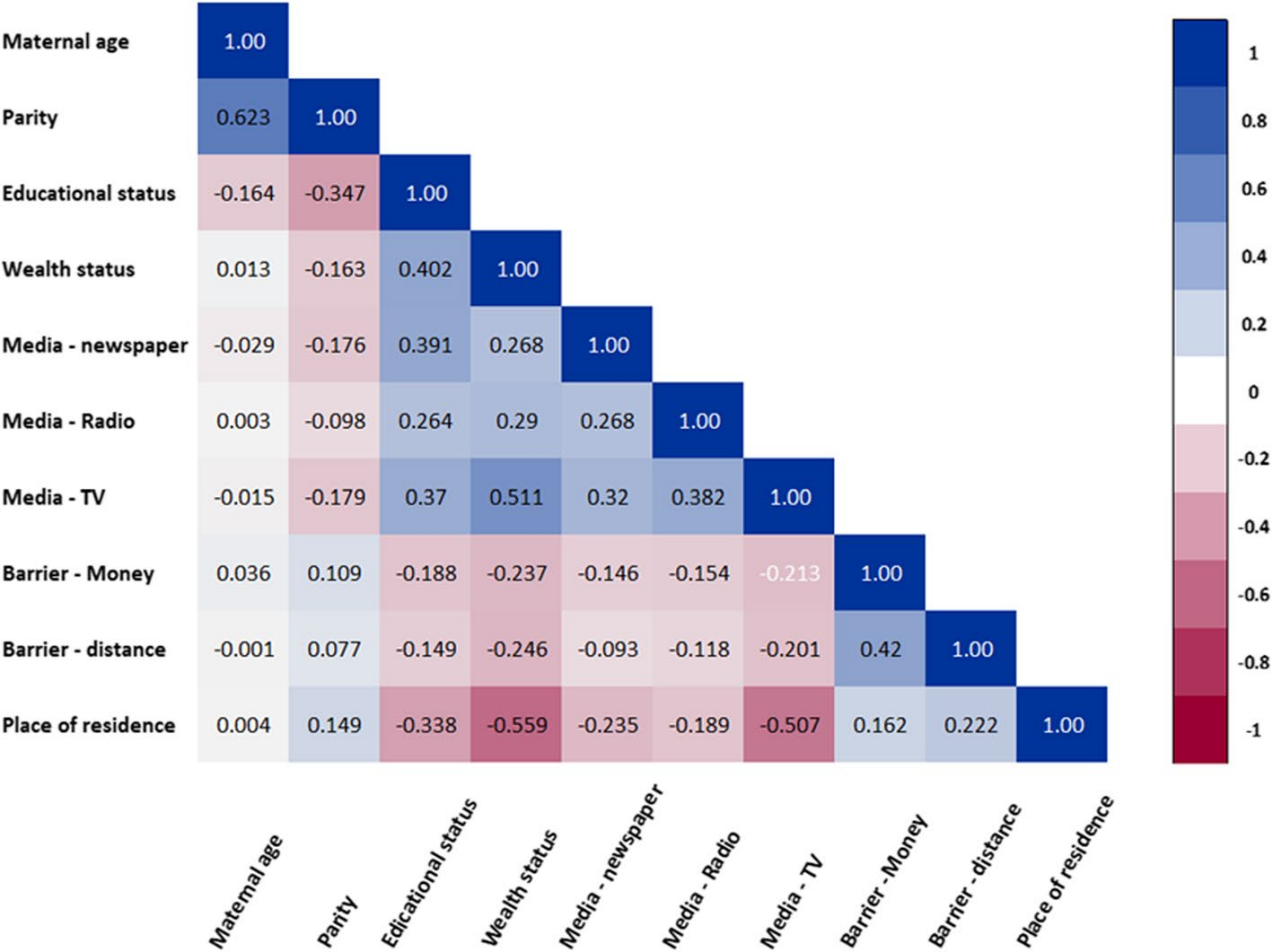
Correlation matrix Note: The correlation coefficient ranges from -1 to 1, where -1 indicates a perfect negative correlation, 1 indicates a perfect positive correlation, and 0 indicates no correlation. Collinearity is typically deemed severe when predictor variables have a correlation coefficient greater than 0.7, as this is assumed to seriously disrupt model estimation and prediction [46]

To reduce the risk of statistical bias, we double-checked our results using univariable analysis and backward elimination analysis. Potential explanatory variables were tested in the univariable analysis and variables with a P-value less than 0.20 were included in the backward elimination analysis. We also tested all potential explanatory variables using the backward elimination method. We assessed collinearity among study factors by examining the variance inflation factor, but no evidence of collinearity was detected in our analyses. The correlations among covariates have also been presented using a correlation matrix (Fig. 1). Results are presented with adjusted odds ratios (ORs), and statistical significance was declared at *P*-value < 0.05. Analyses were conducted using Stata 16.1 and IBM Statistical Package for Social Sciences (SPSS, Chicago, IL, USA) version 27.0.

## Results

### Coverage of maternal health services

**Fig. 2:**
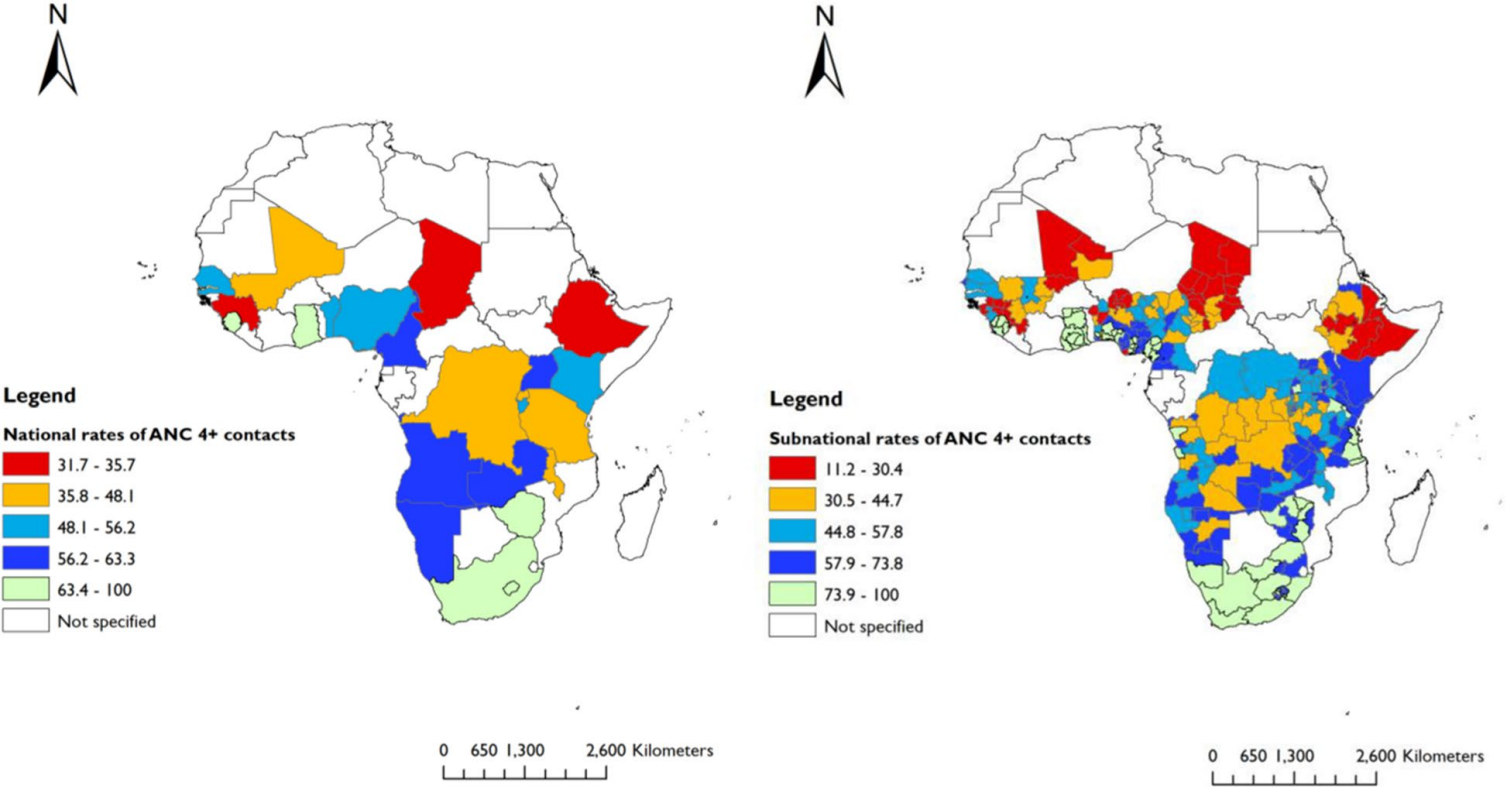
National and subnational rates of four or more ANC contacts in 25 sub-Saharan African countries (DHS 2013 to 2019)

**Fig. 3:**
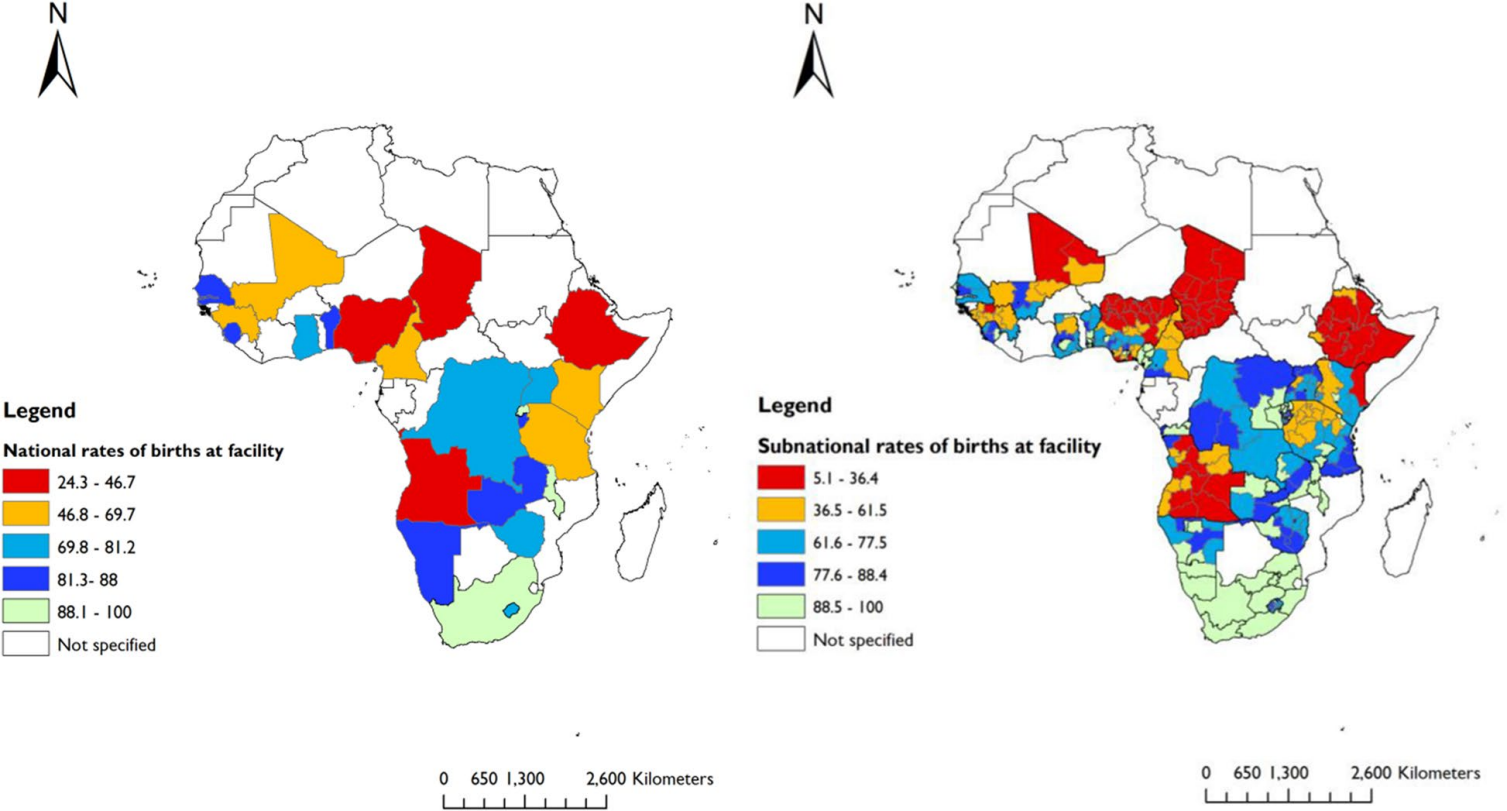
National and subnational rates of skilled care at birth in 25 sub-Saharan African countries (DHS 2013 to 2019)

**Fig. 4:**
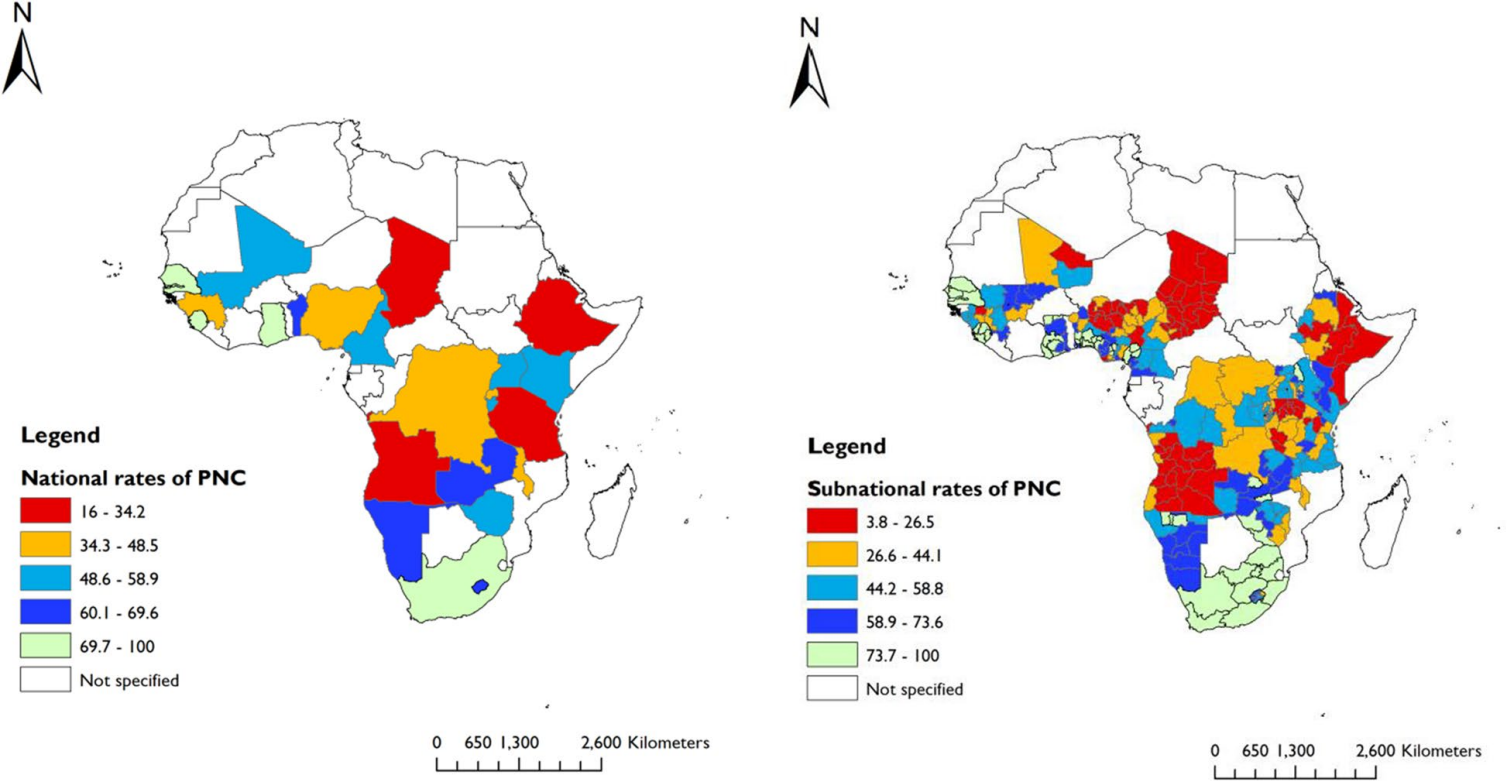
National and subnational rates of postnatal care in 25 sub-Saharan African countries (DHS 2013 to 2019)

We included data on 196,717 women who had a live birth (latest) five years preceding the surveys. The majority (87%) of women had at least one ANC (ANC 1 +) contact, 56% had ANC 4 + contacts, 70% had skilled care at birth, and 48.2% had PNC within the first two days after delivery. In Sierra Leone, women had the highest (89.5%) rates of ANC 4 + contacts, while Chad reported the lowest (31.7%) rates (Fig. 2). SBA ranged from 21.9% in Chad to 95.9% in South Africa (Fig. 3). The rate of PNC was highest in Gambia (87.9%) and lowest in Ethiopia (16.5%) (Fig. 4).

Over one-third of women (33.2%, 95% CI: 32.70% to 33.70%) had an early ANC contact. The proportion varied across countries, with the Democratic Republic of Congo having the lowest rate at 17%, and Ghana having the highest rate at 61%. Slightly more than two-fifths of women (43%, 95% CI: 42.4% to 43.7%) reported receiving all five essential ANC services, which include iron supplements, blood pressure measurement, urine sample test, blood sample test, and tetanus protection at birth. This ranged from 9.2% in Burundi to 68% in Sierra Leone. Nearly nine out of ten women (87.3%) reported having their blood sample collected, 84.6% had their blood pressure taken, 67% had their urine sample tested, and 83% took iron supplements, whereas only 50% reported taking medications for intestinal parasites during ANC contacts.

### Continuum of care coverage

**Fig. 5:**
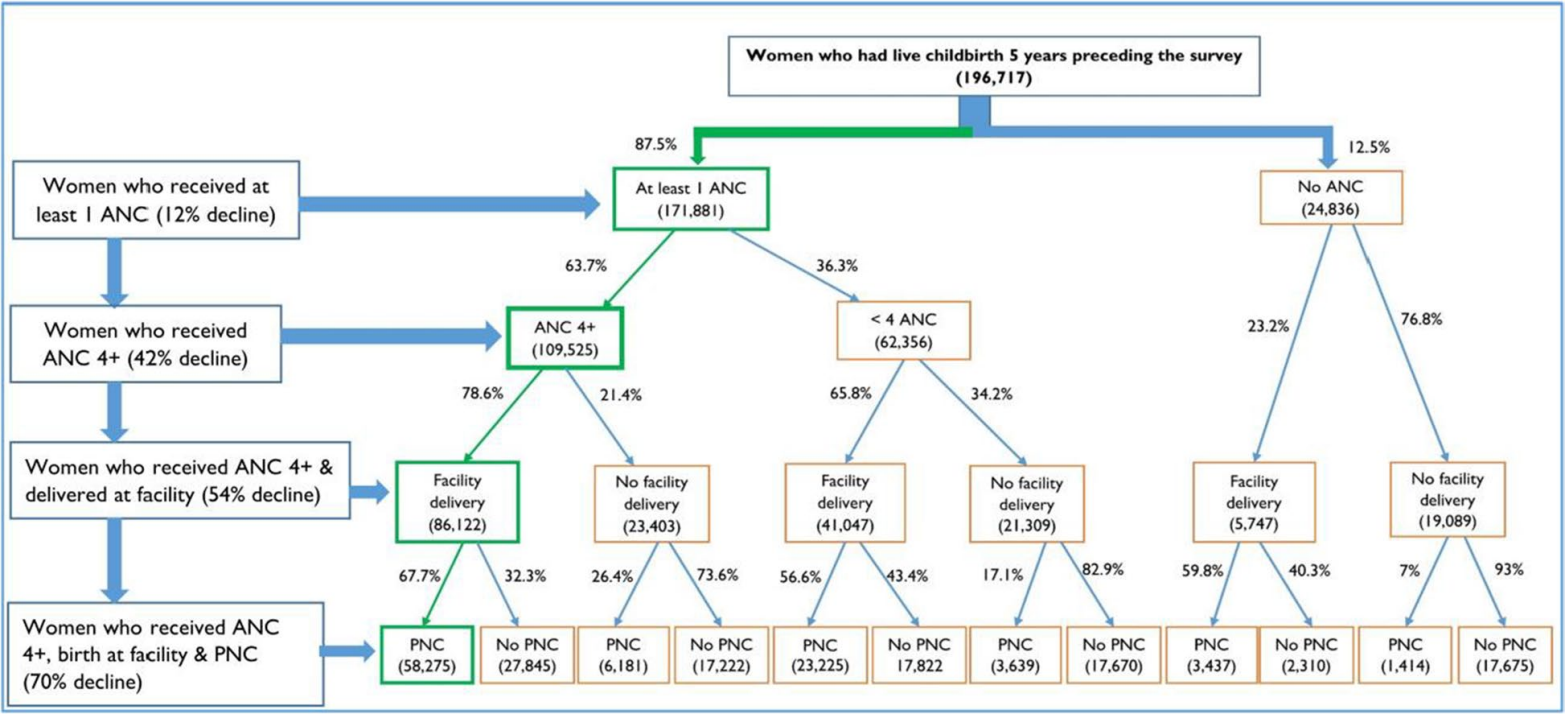
Maternal health care pathway. Green boxes and arrows show the recommended pathway. Rates of decline were calculated for all women (196,717) included in the study (DHS 2013 to 2019)

**Fig. 6:**
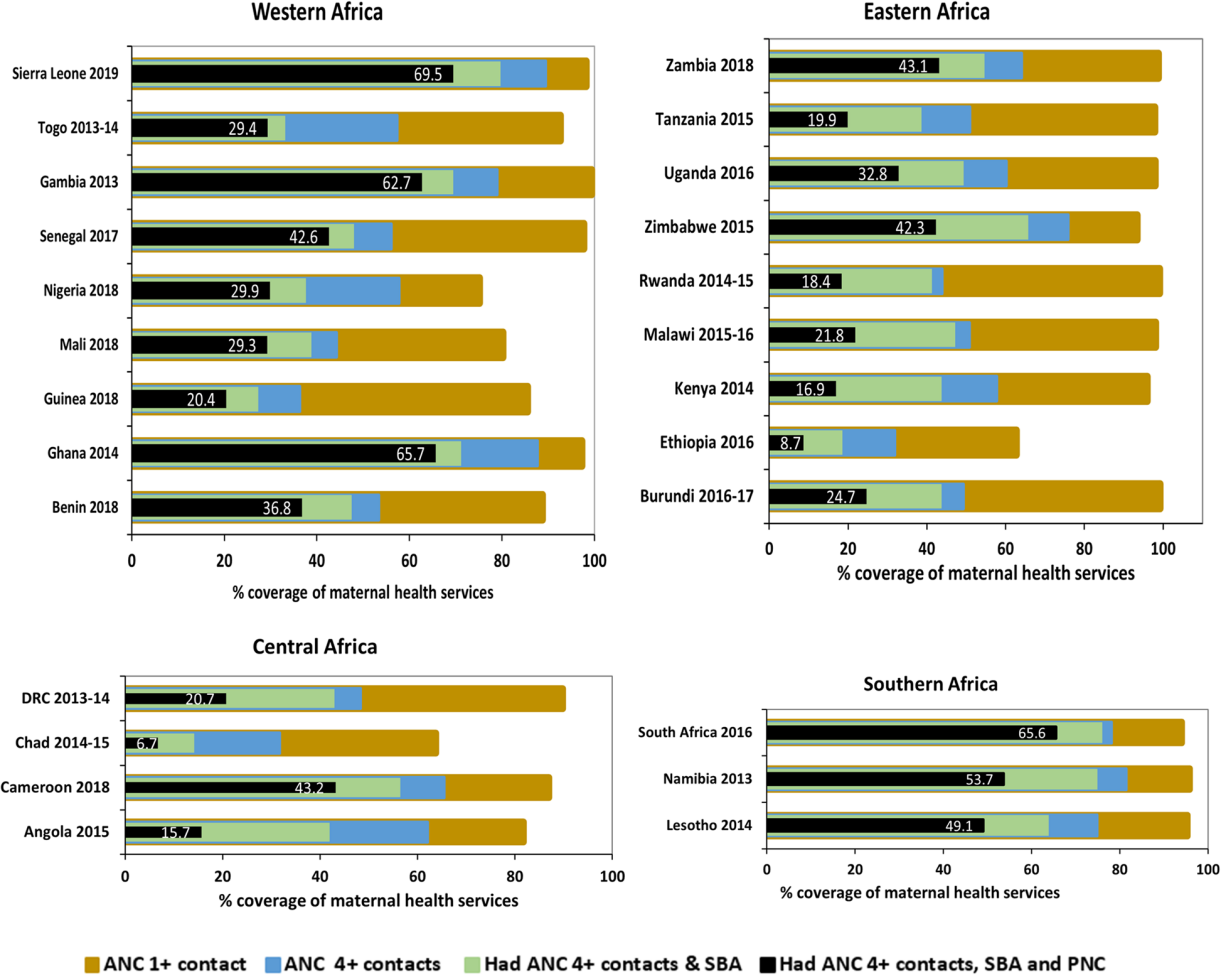
Continuum of care coverage by country – proportions of women who had ANC 1 + contacts, ANC 4 + contacts, those who had 2 services including ANC 4 + contacts and SBA, and those who had completed the continuum of care buy receiving ANC 4 + contacts and SBA and PNC (DHS 2013 to 2019)

A little less than half of women (44.6%, 95% CI: 44% to 45%) reported having both ANC 4 + contacts and skilled care at birth. Four in nine (44.6%, 95% CI: 44 to 45.2) women had ANC 1 contact, skilled care at birth and PNC within the first two days of delivery. Only 29.8% (29.2% to 30.3%) of women received the recommended care package that included ANC 4 + contacts, skilled care at birth, and PNC (Fig. 5). The proportion of women who completed the continuum of care varied by country; the highest rates were registered in Sierra Leone (69.5%) and the lowest in Chad, 6.5%. Namibia (53.4%), Gambia (52.1%), Ghana (65.2%) and South Africa (65.4%) also reported relatively high rates of care coverage across the continuum (Fig. 6).

Along the continuum of care, the uptake of services declined. Continuum of care dropout rates ranged from 30.5% in Sierra Leone to 93.5% in Chad. About one in eleven women (8.5%, 95% CI: 8.2% to 8.9%) reported not having any contact with the health system at any point during pregnancy or childbirth; this ranged from 0.1% in Burundi to 34% in Chad. The rates of women who reported not having care at any stage were also high in Mali (12%), Angola (15%), Nigeria (21.3%) and Ethiopia (33.5%).

### Inequalities in the continuum of care

The wealth-related absolute inequality for all countries across the continuum of care was extremely high, with 29 percentage points between the poorest quintile (18.4%) and the richest quintile (47.4%). The differences exceeded 50 percentage points in countries such as Ghana (50.4%), Togo (54.8%), Cameroon (57.7%), and Nigeria (60.1%).

**Fig. 7:**
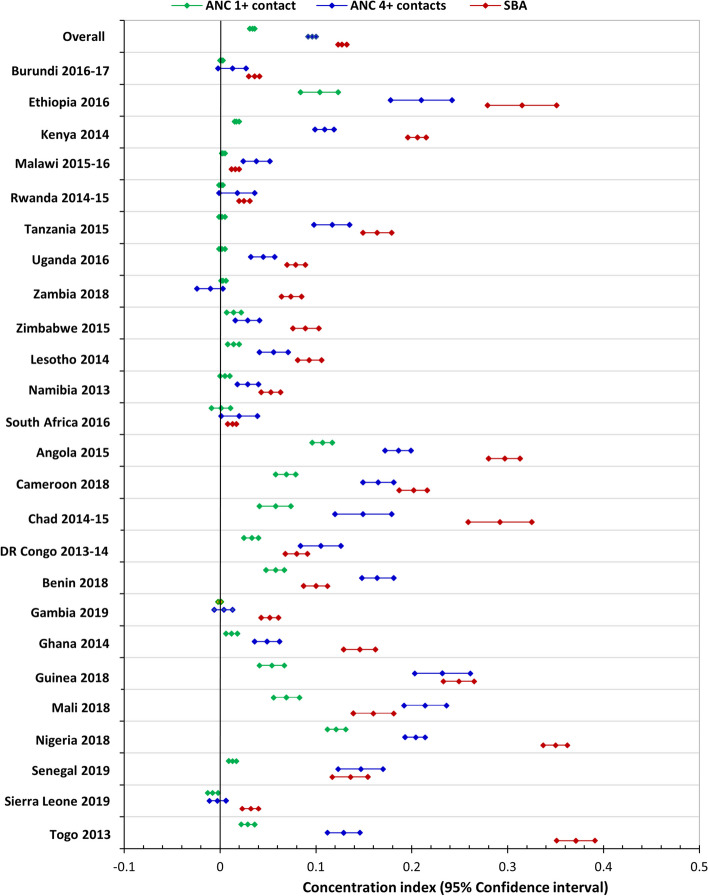
Concentration indices for women who had ANC 1 + contacts, ANC 4 + contacts, and SBA across 25 sub-Saharan African countries (DHS 2013 to 2019)

**Fig. 8:**
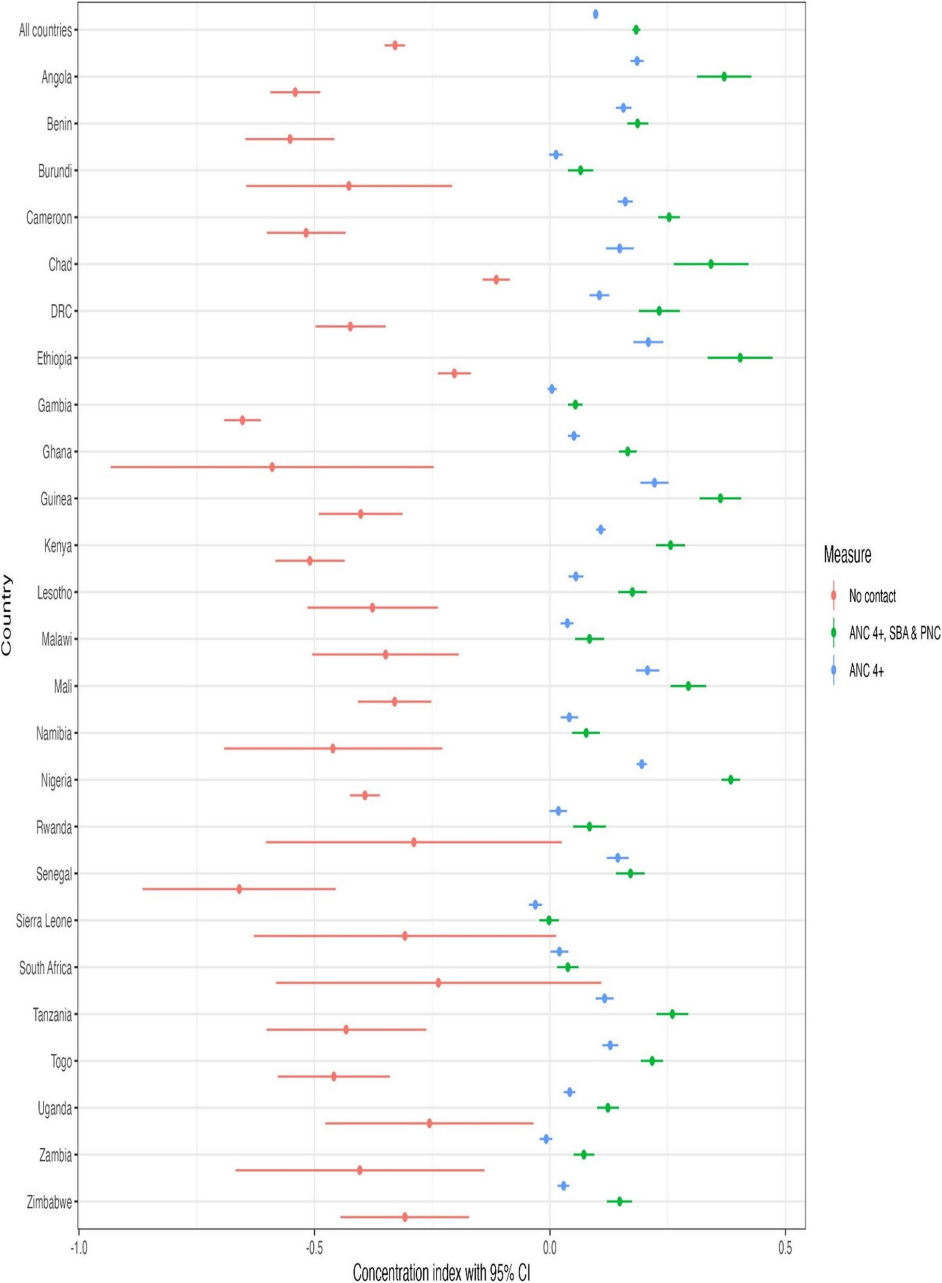
Concentration indices for women who had zero contact, ANC 4 + contacts, and ANC 4 + contacts, skilled care at facility and PNC across 25 sub-Saharan African countries (DHS 2013 to 2019)

Figures 6 and 7 shows inequalities in coverage of maternal health services along the continuum of care. A positive CCI indicates a disproportionate coverage among the rich, while negative concentration indices suggest a disproportionate coverage among the poor. Figure 7 shows that inequalities in utilization of maternal health services increased at each point of care along the continuum of care – we found that ANC 1 + contact had higher coverage and lower inequalities compared to ANC 4 + contacts, while coverage of SBA showed strong inequalities compared to the ANC 4 + contacts across all countries. Figure 8 shows concentration indices for women with zero contacts, ANC 4 + contacts, and women who received all the recommended care packages. In many countries, we observed persistent and increasing inequalities along the continuum of care. Poor women were more likely to have no contact with skilled providers during pregnancy and childbirth than richer women. Having no contact with the healthcare system was disproportionately concentrated among poor women.

We found inequalities in the coverage of ANC 4 + contacts in all countries except Burundi, Gambia, and Zambia, where we found no wealth-related differences. In Sierra Leone, coverage of ANC 4 + contacts (CCI = -0.031) was disproportionately concentrated among the poor, but Guinea reported the highest pro-rich coverage (CCI: 0.222). We also observed strong pro-rich coverage in Angola, Nigeria, and Ethiopia. Inequalities along the continuum of care were highest in countries such as Angola (CCI = 0.37), Chad (CCI = 0.342), Guinea (CCI = 0.362), Nigeria (CCI = 0.384) and Ethiopia (CCI = 0.404).

Table 1 shows predictors of inequality in the continuum of care. Older age women, higher levels of education, exposure to media (newspaper, radio, or TV), and higher household wealth status showed positive associations with the continuum of care. Compared with women aged 15 to 24 years, being in any of the older age groups of 25 to 29 (AOR 1.28, 95% CI: 1.21, 1.36), 30 to 34 (AOR 1.58, 95% CI: 1.44, 1.74) and 35 to 49 (AOR 1.81, 95% CI: 1.54, 2.12) had increased the likelihood of completing the continuum of care. The odds of completing the continuum of care were higher among women with secondary or higher education (AOR 1.77, 95% CI: 1.46, 2.15), followed by women who had primary education (AOR 1.36, 95% CI: 1.16, 1.59) compared to women who had no education. Reading a newspaper at least once a week increased the odds (AOR 1.23, 95% CI: 1.11, 1.38) of completing the continuum of care compared to women who did not read a newspaper at all. The odds of receiving care across the continuum coverage increased with increasing levels of household wealth status: as women of the richest quintile had the highest odds of attendance (AOR 1.87, 95% CI: 1.39, 2.52), followed by those in the third quintile (Q4) (1.52, 95% CI: 1.21, 1.91) and Q3 (AOR 1.29, 95% CI: 1.10, 1.51) than women from the poorest household.

**Table 1:** factors associated with the uptake of care across the continuum

Variables	Frequency	Percent	Percent with all maternal care services % (95% CI)	Unadjusted OR (95% CI)	*P*	Adjusted OR (95% CI)	*P*
**Maternal age**					< 0.001		< 0.001
15–24	57,563	29.3	27.6 (27, 28.2)	1.0 (Reference)		1.0 (Reference)	
25–29	51,040	25.9	30.3 (29.6, 31.0)	1.05 (1.01, 1.09)		1.28 (1.21, 1.36)	
30–34	40,220	20.4	31.5 (30.7, 32.3)	1.08 (1.02, 1.14)		1.58 (1.44, 1.74)	
35–49	47,894	24.3	29.7 (29, 30.5)	0.98 (0.93, 1.04)		1.81 (1.54, 2.12)	
**Parity**					< 0.001		< 0.001
First birth	41,745	21.2	35.7 (34.9, 36.5)	1.0 (Reference)		1.0 (Reference)	
Second birth	37,319	19	32.7 (32, 33.5)	0.85 (0.81, 0.90)		0.79 (0.74, 0.85)	
Third birth	31,479	16	31.4 (30.6, 32.2)	0.82 (0.77, 0.87)		0.71 (0.65, 0.78)	
Fourth birth	25,161	12.8	29.2 (28.4, 30.1)	0.75 (0.70, 0.80)		0.63 (0.57, 0.70)	
Sixth birth	19,590	10	27.1 (26.2, 28.0)	0.71 (0.66, 0.76)		0.59 (0.52, 0.66)	
6^th^ or higher births	41,422	21.1	20.8 (20.1, 21.4)	0.59 (0.53, 0.65)		0.48 (0.40, 0.56)	
**Highest educational level**					< 0.001		< 0.001
No education	66,815	34	20.3 (19.6, 21)	1.0 (Reference)		1.0 (Reference)	
Primary	69,861	35.5	24.9 (24.3, 25.4)	1.56 (1.32, 1.85)		1.36 (1.16, 1.59)	
Secondary	60,038	30.5	45.5 (44.7, 46.3)	2.80 (2.20, 3.56)		1.77 (1.46, 2.15)	
**Wealth status**					< 0.001		< 0.001
Poorest	42,111	21.4	18.2 (17.5, 19)	1.0 (Reference)		1.0 (Reference)	
Poorer	41,369	21	22.9 (22.2, 23.6)	1.31 (1.18, 1.47)		1.17 (1.07, 1.29)	
Middle	39,080	19.9	27.8 (27, 28.6)	1.64 (1.32, 2.04)		1.29 (1.10, 1.51)	
Richer	38,234	19.4	35.3 (34.4, 36.3)	2.41 (1.75, 3.31)		1.52 (1.21, 1.91)	
Richest	35,923	18.3	46.6 (45.5, 47.7)	4.04 (2.76, 5.92)		1.87 (1.39, 2.52)	
**Frequency of reading newspaper**					< 0.001		< 0.001
Not at all	164,995	83.9	27.5 (26.9, 28)	1.0 (Reference)		1.0 (Reference)	
Less than once a week	19,029	9.7	39.7 (38.6, 40.7)	1.65 (1.48, 1.84)		1.15 (1.06, 1.25)	
At least once a week	12,573	6.4	42.9 (41.4, 44.3)	2.02 (1.78, 2.29)		1.23 (1.11, 1.38)	
**Frequency of listening to the radio**					< 0.001		< 0.001
Not at all	85,569	43.5	22.2 (21.6, 22.8)	1.0 (Reference)		1.0 (Reference)	
Less than once a week	38,844	19.8	34.5 (33.6, 35.5)	1.37 (1.26, 1.49)		1.13 (1.05, 1.22)	
At least once a week	72,225	36.7	35.8 (35.1, 36.4)	1.64 (1.51, 1.79)		1.23 (1.16, 1.31)	
**Frequency of watching television**					< 0.001		0.002
Not at all	121,943	62.1	21.8 (21.2, 22.3)	1.0 (Reference)		1.0 (Reference)	
Less than once a week	25,397	12.9	36.2 (35.1, 37.3)	1.51, (1.33, 1.70)		1.11 (1.02, 1.20)	
At least once a week	49,181	25	45.8 (44.9, 46.7)	2.27 (1.94, 2.64)		1.25 (1.13, 1.39)	
**Concerns of lack of money for treatment**					< 0.001		0.011
Not a big problem	85,586	47.1	37.4 (36.7, 38.2)	1.0 (Reference)		1.0 (Reference)	
Big problem	96,263	52.9	26.8 (26.1, 27.4)	0.71 (0.66, 0.76)		0.91 (0.86, 0.97)	
**Concerns of distance to a health facility**					< 0.001		0.007
Not a big problem	112,044	61.6	36.6 (36.0, 37.3)	1.0 (Reference)		1.0 (Reference)	
Big problem	69,802	38.4	24 (23.3, 24.7)	0.75 (0.69, 0.81)		0.90 (0.83, 0.98)	
**Place of residence**					< 0.001		0.001
Urban	65,269	33.2	43 (42, 43.9)	1.0 (Reference)		1.0 (Reference)	
Rural	131,448	66.8	23 (22.4, 23.6)	0.37 (0.28, 0.48)		0.72 (0.62, 0.85)	

We found that factors such as lack of money to pay for treatment, a longer travelling distance to the health facility, and living in rural areas lowered the odds of completing the continuum of care. Women who had financial problems accessing facilities had 9% (AOR 0.91, 95% CI: 0.86, 0.97) lower odds of receiving care across the continuum than women who had no financial problems. Women who traveled long distances to facilities had 10% lower odds of receiving care (AOR 0.90, 95% CI: 0.83, 0.98) compared to women who *did not* travel long distances to facilities for care. Women from rural areas had 28% lower odds of receiving care across the continuum (AOR 0.72, 95% CI: 0.62, 0.85) than women from urban area.

## Discussion

Continuity in the use of ANC, SBA, and PNC substantially improves MCH outcomes [12]. However, the use of these services is either limited and/or fragmented in many sub-Saharan African countries. In our study, 87% of women initiated contact with the health system during pregnancy, but only 30% of women completed the continuum of care.

Over half of all maternal and newborn deaths occur during birth and the first few days of life [21]. During these early days, when mothers and newborns need care and have a higher risk of dying than at any other time, access to care is limited. In our study, only 48% of women reported receiving PNC within the first two days. This could be associated with countries' MCH programs, which tend to prioritise improving the rates of SBA rather than other MCH services [13]. We found higher rates of SBA compared to ANC 4 + contacts, and coverage of care was at its lowest during the postpartum period.

We found geographic and wealth-related inequalities within and across countries. Geographic inequalities explain the poor access and low healthcare uptake commonly observed across less developed regions characterized by limited health facilities, schools, electricity, and road infrastructure. Low coverage is also common among vulnerable and hard-to-reach communities [22].

We observed significant dropouts along the continuum of care. This finding is consistent with previous studies [23–25]. Gaps in the continuity of care from ANC1 to ANC4 + contacts might be because of delayed initiation and poor content of care women received during early contacts. The majority of women (63%) had delayed ANC initiation. Women also received low content of care across all countries and socioeconomic levels. Even when women managed to have adequate ANC contacts, nearly half of women reported receiving blood sample tests, blood pressure taken, urine sample test, iron supplements, and tetanus protection at birth in the current study. This shows significant gaps in the quality of ANC women received. This is also in line with previous studies in the region [26]. WHO now recommends a minimum of eight contacts in the new ANC model introduced in 2016, but increasing the number of contacts alone cannot improve health outcomes without improving the quality of care delivered to women [27].

Substantial gains in maternal health can be made by improving access to care during the postpartum period [2, 28]. However, health facilities and providers give less attention to postpartum care. The hours and days after delivery remain a critically dangerous period for both mother and newborn [13, 29]. During this period, more than 60% of all maternal deaths occur, and the death of newborns amounts to 47% of all child deaths under the age of 5-years [1]. This period is where the continuum of care is most often interrupted. Even when deliveries occur at healthcare facilities, many mothers and babies are discharged early, sometimes within a few hours [13, 30]. Our findings also showed low coverage of PNC and high dropout between delivery and the postnatal period. The decline in care could be associated with a lack of a clear line of professional responsibilities [12]. In many countries, the handover and communication between maternal and child health programs are not well established [2].

The overall coverage of the continuum of care was low across countries and socioeconomic levels. Women of low socioeconomic status require affordable, acceptable, and appropriate access to quality health services as they bear the greatest burden of morbidity and mortality [2, 31]. However, this is not always the case, as poor women and children have poorer access. In our analysis, the rates of no contact were higher among the poorest by 14% compared to women in the richest quintile. The rates of women who have not had any care during pregnancy and childbirth were more than 35% among the poorest quintile in countries such as Chad (39%), Angola (40%), Nigeria (43%), and Ethiopia (50%), but this was less than 1% among the richest quintile in 20 of the 25 study countries. The gap in having a full continuum of care was also large, with a difference of 29 percentage points between the poorest quintile (18.4%) and the richest quintile (47.4%).

Overall, countries such as Nigeria, Ethiopia, Chad, Guinea, Angola, and the Democratic Republic of Congo experienced lower coverage and higher inequalities in providing maternal health services across the continuum of care. These countries are also known for having weak healthcare delivery systems [32]. In 2018, the performance of those countries in terms of access and quality of care was among the poorest globally. According to a study evaluating health system performance based on access and quality of care, Nigeria ranked 142^nd^, Angola (162^nd^), Democratic Republic of Congo (181^st^), Ethiopia (184^th^), Guinea (190^th^), and Chad (192^nd^) out of 195 countries. Furthermore, a World Bank report indicated that these countries also scored lower on the universal health coverage index. Most sub-Saharan African countries had universal health coverage (UHC) index below 45; for example, Nigeria had a UHC index of 42, Angola 39, Ethiopia 38, the Democratic Republic of Congo 39, Guinea 37, and Chad 28, highlighting a severe shortage of essential healthcare services in these regions [33]. In contrast, countries with stronger economies and more established health systems, such as South Africa, Ghana, Kenya, and Rwanda, more recently consistently exhibited higher coverage and lower within-country inequalities in accessing quality MCH services [32].

The fact that almost all women (87%) initiated contact with the health systems presents an opportunity to strengthen maternal, neonatal and child health. This may be accomplished through the delivery of high-quality essential interventions that include ANC, skilled care at birth, PNC, and related neonatal and child health services. However, women in many sub-Saharan African countries encounter multiple challenges that limit their access to these services. Poor implementation of service packages, inadequate linking of services in the care package, and neglect of some critical interventions could contribute to access constraints [2, 13]. Delays in seeking care, financial hardships, and poor-quality care in health facilities also contribute to poor MCH outcomes [12, 29].

Time and again, evidence has shown that poor quality of care is the most hindering challenge to improving maternal health services. Improving access alone will not bring the required change in maternal health. The findings of our study also indicate that poor quality of care may have contributed significantly to the high dropout rate from the continuum of care. Clearly, the large majority (87%) of women had access, but they were not able to complete the continuum of care. This shows how health improvements can remain elusive even with increased and equitable access to services unless those services are of sufficient quality to be effective. This also implies that quality improvement initiatives that only focus on the population at large, but fail to address vulnerable populations, can result in unequal quality. Thus, improving maternal health requires action to ensure that all women have access to high-quality maternal health care, regardless of their socioeconomic status or where they live. This includes providing access to care for those who are currently outside the system.

Several factors contribute to inequalities in MCH services. Older age women, women with lower parity, those who had primary or higher education, women with improved access to mass media, and those from higher wealth status had higher odds of completing the continuum of care.

The influence of maternal education and exposure to media on access to maternal healthcare services could be due to the role education and media play in bridging the knowledge gap by informing and sensitizing women on the benefits of MCH services, which leads to positive attitudes and improves health-seeking behavior. As a result, women with higher education and exposure to mass media were more likely to initiate ANC early and complete the continuum of care in our analysis. This finding is consistent with previous studies in the area [23, 24, 26, 34].

In our study, women who travelled long distances to health facilities, lacked money for treatment, and lived in rural areas were less likely to complete the continuum of care. Women and children in rural areas, marginalised groups and those living in hard-to-reach and remote areas are the most underserved [35–37]. For example, women in rural sub-Saharan African countries were less likely to receive skilled care at birth than their urban counterparts [34]. However, disadvantaged women in urban areas do not always have better access, highlighting that poverty is one of the main drivers of inequality in the use of healthcare services. For example, caesarean rates were the lowest among the poorest women in rural and urban areas in many sub-Saharan African countries [34].

Inequity also manifests in other ways. Many women experience mistreatment while attending health facilities for pregnancy and childbirth [38, 39]. This is even more common among poor women. For example, in many sub-Saharan African countries, poor women experience verbal and physical abuse from providers in many health facilities [40, 41]. Lack of respectful care discourages women from using available services and erodes public trust in the health care systems [38].

Strengths of our study include the use of nationally representative population-based survey large datasets across multiple sub-Saharan African countries. We used multiple approaches to examine inequalities along the continuum of care for maternal health services. The limitations of our study include using data from the DHSs based on a recall period of 5 years. The latest DHS datasets for some countries date back to 2013, and there may be differences in the current rates for some countries. Another potential limitation of the study is that maternal deaths occurring along the care pathway were not taken into account, as the data from the DHS only included information on living women.

### Policy considerations

Adapting and implementing the continuum of care as a priority strategy effectively achieves high coverage of maternal, neonatal and child health interventions [12]. Thus, to improve health and achieve the greatest reduction in deaths and morbidity, all of these packages must reach mothers and their children at the appropriate level and time period. To that end, we propose the following policy recommendations.

First, improve access along the continuum of care by strengthening the link between place and time of services to reduce dropouts [2, 12, 13]. Our analysis revealed that the poorest women living in rural areas and those who had no education or access to media had the highest attrition along the continuum of care. Addressing the needs of underserved women would require linking home and community care to quality healthcare services at primary health facilities and district hospitals, which is crucial to bringing care closer to women and women closer to care.

An example of an effective intervention is the establishment of maternal waiting homes, community-based facilities situated near health centers or hospitals, allowing pregnant women to stay close as their due dates approach, thereby reducing delays in accessing care during labor and delivery. Another important measure to reinforce these linkages is through Birth Preparedness and Complication Readiness (BP/CR) Programs, which educate families and communities about the significance of preparing for childbirth, identifying danger signs, and knowing where to seek timely care in case of complications. Establishing or strengthening these ties would increase interactive dialogue within communities, improving service use. These linkages will also help that women and newborns with complications are referred promptly and receive appropriate care at a proper level to improve their survival rate.

Second, improving the quality of care across the continuum of care increases service uptake. Addressing poor quality of care has been a critical challenge to health systems worldwide [42]. Poor quality of care is a known cause of delay for women and children in seeking care, more so for poor and marginalized groups, who might receive a service that is not only technically inadequate but also violates their right to respectful treatment at times [38]. Disrespectful care is also a known deterrent to women from having subsequent contact with the health systems.

Third, improve access for vulnerable populations to address low coverage. Equity-oriented interventions are vital to target and address the needs of specific groups based on their poverty level, geographical location, and other factors that characterize vulnerability [14, 43].

Fourth, provide economic support for vulnerable women through funding for transport infrastructure and subsidies for service users to encourage service uptake. Conditional cash transfer works by transferring cash to poor households and other vulnerable groups to stimulate the uptake of health interventions [44]. Evidence suggests that targeted, conditional cash transfer programs can improve the use of healthcare facilities by the poorest women and children [45].

## Conclusion

Coverage of continuum of care was low across all countries and socioeconomic levels. We found persistent and increasing inequalities along the continuum of care for maternal health services as more socioeconomically disadvantaged women dropped out of care during pregnancy, childbirth, and the postnatal period. Disadvantaged women were more likely to have no contact with the healthcare system and less likely to access the continuum of care than wealthier women. Women who received the continuum of care were educated, wealthy, urban residents who accessed mass media and had no distance problems accessing a health facility. Higher integration and improved access to quality healthcare services are required to enhance women's and children's health outcomes. Initiatives to improve maternal health services should prioritize the needs of communities and groups with low maternal health service coverage.

## Data Availability

This study was based on an analysis of existing dataset in the DHS repository that are freely available online with all identifier information removed (http://www.dhsprogram.com).
